# Factors Affecting Treatment Regress and Progress in Forensic Psychiatry: A Thematic Analysis

**DOI:** 10.3389/fpsyt.2022.884410

**Published:** 2022-07-12

**Authors:** Riitta Askola, Olavi Louheranta, Allan Seppänen

**Affiliations:** ^1^Department of Psychiatry, University of Helsinki and Helsinki University Hospital, Helsinki, Finland; ^2^Department of Nursing Science, Faculty of Health Sciences, University of Eastern Finland, Kuopio, Finland; ^3^Niuvanniemi Hospital, Kuopio, Finland; ^4^Vanha Vaasa Hospital, Vaasa, Finland

**Keywords:** forensic psychiatric services, deinstitutionalization, transinstitutionalization, reform of mental health legislation, national quality standards

## Abstract

International variability and shifting trends in forensic psychiatry lead to gaps in national service provision and needs for service development. This study explores these needs through the subjective narratives of those involved in Finnish forensic services, either as forensic psychiatric patients, their parents, or service providers. Data was gathered by means of thematic interview and subjected to thematic analysis. Three main themes emerged: (1) pre-treatment challenges, (2) institutional/treatment-related concerns about therapeutic security and (3) adapting and recovery. The research highlights the need to develop forensic psychiatric services at three levels. First, it calls for increased risk awareness and risk assessment skills at the general psychiatric level. Second, it emphasizes the need for increased therapeutic engagement throughout the rehabilitative process. Third, it calls for structured and meaningful post-discharge aftercare. At all three levels, gradated security-aware standardization and patient triage in forensic services would help to develop and maintain an intact care pathway. This would decrease offending, marginalization, and suffering. Only then can we begin to meet the requirements of the WHO European Mental Health Action Plan. These findings can contribute to the development of international, standardized treatment models for clinical forensic psychiatric practices.

## Introduction

The World Health Organization's European' Mental Health Action Plan 2013–2020 (WHO action plan) (1) strives to (a) improve the mental wellbeing of the population and reduce the burden of mental disorders, with a special focus on vulnerable groups, exposure to determinants and risk behaviors, (b) respect the rights of people with mental health problems and offer equitable opportunities to attain the highest quality of life, whilst addressing stigma and discrimination, (c) establish accessible, safe, and effective services that meet people's mental, physical, and social needs and the expectations of people with mental health problems and their families. To this end, the WHO proposes a reorganization of mental health services, including continuing psychiatric deinstitutionalization and increased provision of easily accessible local services.

Implementing the WHO proposals is not yielding the expected results. For instance, while the overall number of general psychiatric hospital beds has indeed decreased, the number of places in other institutions, including prisons, forensic units and supported housing has increased, in an apparent process of transinstitutionalization (2, 3). In Western Europe from 1990 to 2000, the number of psychiatric hospital beds decreased on average by 42.5 beds per 100 000 inhabitants, and from 2000 to 2012 the decrease was 22.44; at the same time, forensic beds rose by an average of 0.49 between 1990 and 2000 and 0.76 between 2000 and 2012 (2). Suggested reasons for the increase in the number forensic psychiatric beds, in addition to the decrease in general psychiatric bed provision, include an increase in comorbid substance abuse, increased risk aversion in society (4), and the loss of social support for mentally ill people in traditional families (5). Whatever the specific determinants prove to be, these trends have nonetheless caused a significant strain on psychiatric services (6, 7) – institutional or otherwise – and a need, in many countries, to reassess the role, quality, and organization of forensic services (8–11).

Forensic mental health services (FMHSs) are primarily designed to provide treatment in conditions of therapeutic security for persons with severe and often disabling mental disorders and offending behaviors (12). Thus, forensic psychiatric treatment is either an independent medical specialty in some countries, including Finland (13), or a recognized subspeciality in others (14). Specific attributes of forensic treatment include the need for risk awareness and risk assessments in clinical decision making, multifaceted conceptualization of security (15, 16), common presence of clinical comorbidity, effect of legal stipulations arising from patients' offending history, and lengthy treatment periods (17, 18). Institutional FMHSs are high cost, high risk and low volume, and thus must yield high value in health gains and risk management (19, 20).

Despite international attempts to coordinate forensic mental health service organization and legislation, the forensic patient population differs significantly from one country to another even within the European Union (EU). Questions of criminal responsibility, diversion mechanisms from the criminal sanctions agencies and many aspects of involuntary treatment are defined by national legislation, rather than international, evidence-based models of care (21–24). Bed numbers and treatment duration have increased in several EU states, but not in others. Reasons for this may be social, political, and economic, such as a country's GDP and healthcare spending, the relationship between prison places and psychiatric beds (25), cultures of risk containment, familial and community support structures, levels of poverty, and legal frameworks (26).

Over time, forensic services and patient populations change. Degl' Innocenti et al. (27) indicated a significant shift in their register-based study of Swedish forensic patients in 2010 and 2018: from inpatient to outpatient care, from first-generation antipsychotics to second-generation antipsychotics, and to shorter lengths of stay, particularly for men. Use of physical restraints and forced medication diminished while less severe restrictions, e.g., on communication rights, increased.

Finland has a national mental health policy for 2010–2015 (28) and national mental health strategy and suicide prevention program for 2020–2030 (29). Echoing the WHO action plan, the national mental health policy emphasized the development of community services, downsizing institutional care, and the closure of separate mental hospitals whilst strengthening preventive strategies, collaboration between different administrative branches of health and social care providers, and supporting mental health capacity in primary care (28).

Traditionally, in Finland forensic psychiatry has had three main functions: (1) providing forensic psychiatric assessments for courts deciding on criminal responsibility, (2) providing treatment to those offenders who are not sentenced based on criminal irresponsibility, (3) and treating patients who have been transferred from general psychiatric units as their treatment is dangerous or difficult (13, 30). In Finland (population ca. 5, 5 M), ca. 80–100 forensic psychiatric assessments are currently produced per year (down from ca. 300 in previous decades) and about 30–35 offenders are annually committed to involuntary forensic treatment for a median of 5–9 years (13, 31–34). Finland has two state forensic hospitals, university hospital forensic units, and prison psychiatric services. Prison security standards cover prison healthcare units, but Finnish forensic unit security standards, as defined for instance by UK professional bodies and medicolegal authorities (35–37), do not adhere to anything akin to high or medium security. In fact, there are no national standards or legal stipulations whatsoever concerning the physical, operative, and security attributes of forensic services. Instead of preventive action, Finnish medicolegal authorities rely on reactive ex-post monitoring of individual cases.

In the following, we explore the gaps in forensic services in Finland through the subjective narratives of those involved, as patients, carers, or service providers. More specifically, we were interested in which factors in the services affect treatment regress and progress, and, accordingly, how could the services be developed based on these insights.

## Methods

### Participants

Eight forensic psychiatric patients (*n* = 8), the parents of six forensic psychiatric patients (*n* = 6), and nine forensic psychiatric nurses (*n* = 9) from two forensic psychiatric hospitals in Finland were interviewed. The inclusion criteria for the patients were: 1) age over 18, (2) mentally stable enough to participate (i.e., no excessive anxiety anticipated due to participating), and (3) sufficient proficiency in Finnish. The exclusion criteria were mental instability (acutely psychotic, suffering from anxiety, likely to self-harm, or in the personnel's estimation likely to be adversely affected by participating in the proposed study). The patients, all aged 30–50, were either inpatients (*n* = 6), or outpatients (*n* = 2) discharged by the National Institute for Health and Welfare (THL) under supervision and living in psychiatric rehabilitation units. Their index offenses included homicides (four patients), crimes against property (one patient), assaults (two patients) and arson (one patient). Seven patients were men and one was a woman.

The inclusion criteria for the parents/carers were: (1) being a parent of forensic psychiatric patients, (2) willingness to participate in the study, (3) patient's permission to contact the parents. Five of the parents were women, and one was a man.

The inclusion criterion for forensic psychiatric staff participant selection was being a registered nurse (RN) or mental health nurse acting as a named nurse. The sample selection was based on the relevance of the nurses' experience, with all nurses selected having at least 10 years' psychiatric nursing and 5 years' forensic psychiatric nursing experience. Eight of the nurses worked in an inpatient setting, whereas one in an outpatient forensic clinic. Five nurses were men and four were women.

### Procedure

Ethical approval for the study was obtained from the Ethics Committee of the Hospital District of Helsinki and Uusimaa. Formal approval and permission for data collection in the relevant treatment units and from the parents of forensic psychiatric patients were granted. Having obtained permission for data collection the researcher (RA) informed nurse managers and the staff on the wards and out-patient clinics about the study. The staff suggested suitable patients whom they thought would not be distressed by the study and who were in a stable enough condition to participate in relatively lengthy interviews. The nursing personnel assessed the parents of those forensic psychiatric patients whom they estimated would be willing and able to participate in the proposed study. After this assessment, they requested their patients' permission to contact the parents. The nurses informed both the patients and their parents about the study, and if both parties agreed, they passed on the parents' contact information to the researcher. The researcher then contacted the parents with more detailed verbal and written information about the study, after which each parent signed an informed consent form. The researcher had had no part in the treatment of the patients whose parents were to be interviewed.

The interviews were performed independently of each other. The researcher was not previously known to the nurses, or the patients interviewed, or the parents of the forensic psychiatric patients. Also, the nursing staff and parents in this study were not those of the studied patients. Participants were given written and verbal information regarding the study and formal informed consent was obtained from all participants. The researcher (RA) conducted all the thematic interviews herself. Thematic interview was chosen as a method, as it allows acquiring qualitative information about a topic or about a field which is relatively less known or rarely studied. It focuses on subjective experiences as defined and narrated by the interviewees and accepts this as valid material for scientific scrutiny (38, 39).

All interviews were recorded except two, as the patient and the parent concerned objected. For these two interviews, the researcher took notes instead. The participants were asked to describe factors affecting treatment regress and progress in forensic psychiatry in their own words. Some of the guiding questions asked during the interview process concerned challenges in treatment and recovery. All interviews were transcribed verbatim. A total of 194 pages of material (1.5 spacing) resulted. Of these, 98 pages concerned the interviews with patients, 27 pages concerned the interviews with the parents, and 69 pages, the interviews with nurses. The research material was meaningful as it represented authentic experiences and gave the subjects themselves a voice to a sensitive subject.

### Analysis

Inductive thematic analysis was applied to the data. In inductive analysis, the data is coded without using an existing framework and aiming to avoid the analyst's' preconceptions (40). A key aim of the process of coding and thematic analysis is to retain detail in the data items; codes are labels applied to segments of data which are likely to be relevant in the context of the research questions (41). Thematic analysis is suited to analyzing subjective experiences, perceptions, and understandings (41, 42). It can be used to identify, analyze, and report patterns (themes) in qualitative data, particularly for examining the perspectives of different research participants, highlighting similarities and differences, and generating unanticipated insights (40). Therefore, data from the forensic psychiatric patients, their parents, and their nurses, were analyzed together.

The data was analyzed in six phases (40), by utilizing guidelines and tools to support the process of conducting a rigorous and trustworthy thematic analysis (43, 44). First, the researcher listened to the audiotapes and read the transcripts several times to develop a thorough understanding of them. Second, the researcher generated initial codes to identify each feature of the data that appeared noteworthy. The entire data set was organized into groups according to the codes. Coding was done manually and no qualitative data analysis software was used in analyzing the data. Third, the codes were sorted into potential themes, with consideration of how codes could combine in an overarching theme. Fourth, the themes worked were checked against the coded extracts and the data as a whole. A candidate thematic map of the analysis was generated. Fifth, the themes were defined and named, and sub-themes identified. Again the coherence of and relationship between the themes were checked. Sixth, the report was produced after selection of compelling extract examples that related to the research question.

## Results

**Figure 1:**
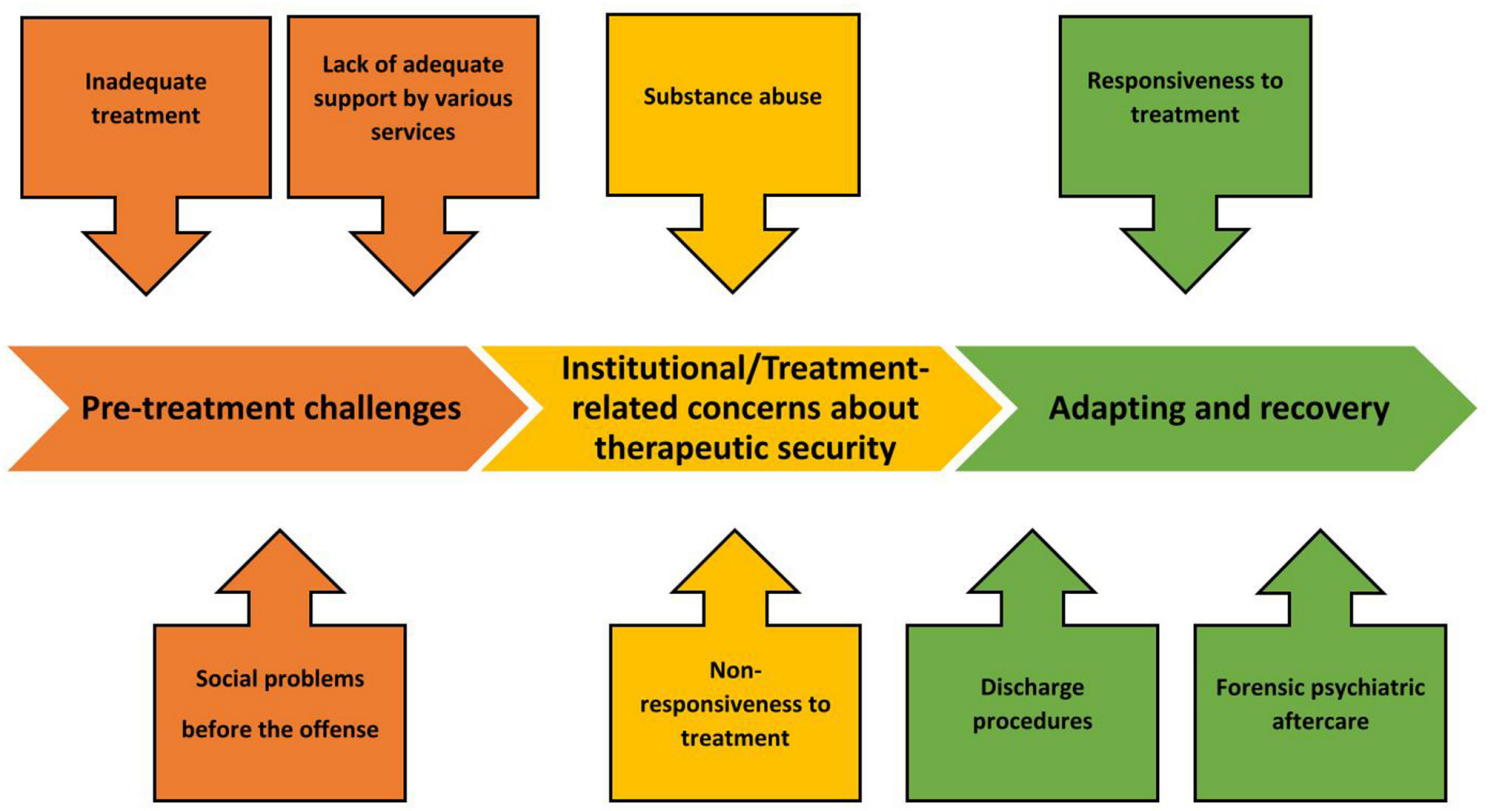
Factors affecting treatment regress and progress along the forensic rehabilitation process. The main themes are (1) pre-treatment challenges, (2) institutional/treatment-related concerns about therapeutic security and (3) adapting and recovery. For each main theme, (1–3) subthemes emerged, as illustrated.

Three main themes emerged from the data: (1) pre-treatment challenges, (2) institutional/treatment-related concerns about therapeutic security, (3) adapting and recovery. Each theme included subthemes. These factors affecting treatment regress and progress in the process of forensic rehabilitation are illustrated in Figure 1.

### Pre-treatment Challenges

This main theme contained three sub-themes: social problems before the offense, lack of adequate support by various services, and inadequate treatment.

Within all three groups of interviewees (patients = P, parents (carers) = C, nurses = N), answers indicated that before the offense patients had considerable social difficulties, such as early-onset offending behavior, social marginalization, substance abuse, criminal recidivism, and prison re-entry.

I was continually in and out of prison, like the judge said in court “You don't really enjoy civilian life, do you?”, which was true, I found it easier in prison. P8

Parents felt they had not received adequate support from the healthcare services and sometimes they had had to deal with the judiciary after their child had assaulted them. Indeed, a recurring theme in the narratives of some parents was being repeatedly victimized by their child but not getting help from the police. In addition to the offense and risk behavior, they were burdened by violence, the stigma of familial mental illness, and the breakdown of family relations.

I told the doctor you really need to get him into treatment, look at how bruised I am. I'm being kicked around. He felt there was not enough evidence for that, not enough on the computer. What more should happen? Not enough indications, or what was it he said? C1

Before the index offense, patients had often been committed to involuntary treatment several times, without receiving adequate treatment. Patients in risk of offending behavior either were not recognized as such or the risk was not managed assertively enough. Parents also felt that they had not been included in the treatment process, or they had been inadequately informed.

He was so intimidating there, so intimidating that they were afraid to keep him there. N9
He was there for only a week, and then into the community. So that didn't work at all. Well, they were supposed to make home visits, but he didn't let them in and they said they hadn't the authority to do anything. C2

### Institutional/Treatment-Related Concerns About Therapeutic Security

This main theme contained two sub-themes: non-responsiveness to treatment and substance use.

The patients' or their parents often had a negative attitude to forensic assessment and treatment. One parent questioned the timing of the forensic assessment, claiming that it was too early with respect to recovery, thus distorting the outcome. One patient said that his prison sentence for the index offense would have been shorter than the treatment order, which he compared to a life sentence.

I think that's total bullshit, I'm not insane. I belong in prison. I would have rather been in prison. If I had been sentenced, I would have been out years ago. Now it takes longer. P1

Being a parent of a forensic patient can be ethically challenging, as parents may possess information on issues that could be valuable in terms of forensic treatment, but could prolong the treatment period or have other unwarranted effects. Parents could also blame the service providers for the index offense and refuse to collaborate.

As a mother she opposes treatment and medication, like, her child doesn't need medication. N9

Treatment progress could be affected by the patient's lack of treatment motivation and engagement, or by a particularly challenging, drug-resistant disorder. One nurse described how his patient had been on electro-convulsive therapy (ECT) and various medications, at maximum doses, without intended clinical results. According to the interviewee, this created the need to resort to various restrictive practices, such as seclusion.

Very ill and drug-resistant in addition to everything else, like new medication trials are done continuously, and ECT was given during the winter… but he is more or less agitated, anxious, and that results in him envisioning how he would stab for instance me or someone else. If he has very intense thoughts about harming himself or his roommate, he can be placed in seclusion. N7

Nursing staff recognized the formation of various subcultures among patients, which centered around substance abuse. These subcultures affected inter-patient dynamics and the ward environment by increasing restlessness and decreasing commitment to treatment measures.

One becomes quite restless, in a way. Is there something going on, are there drugs in the hospital, are there thoughts of escape or is there something you know, that you don't want to know about? There can be all kinds of things going on among the patients, which even the nurses don't necessarily know about. N8

Substance misuse had a negative impact on treatment. According to one nurse, a patient who had progressed well in terms of rehabilitation, was in risk of reverting back to substance misuse and resultant apathy, loss of circadian rhythm, and addiction.

Substance abuse is the worst, there's no interest in studies or anything, they're addicted to drugs and those circles, and they mess about and we can't seem to catch them. N9

The risk of substance abuse was perceived as particularly pressing after discharge, due to its connection with offending behavior.

In the end I became addicted. I think that if I had stayed in hospital, perhaps these things would never have happened. P3

### Adapting and Recovery

This main theme contained three sub-themes: responsiveness to treatment, discharge procedures, and forensic psychiatric aftercare.

Treatment adherence expedited rehabilitation. Particularly, adherence to medication was seen as pivotal.

And I think medication is really important, I'm sure to be using it for the rest of my life, so nothing like this will ever happen again and I can live a so-called normal life and cope with this illness in the future too. P2

Treatment adherence was also affected by the quality of the therapeutic alliance that patients and staff were able to form. Indeed, both staff and patients emphasized the importance of cultivating a trustful and accepting relationship.

You notice, when in conversations your thoughts start being on the same level, that you're forming that connection; then it starts working. P8
The patient starts to understand that we're actually on the same side here. That we're trying to push him out of here, not hold him back, no confrontation. N2

Collaboration with parents, supporting them, and offering psychoeducation, group interventions and peer-support became increasingly important as treatment progressed toward life outside the hospital.

We had one of those anger management groups here. Luckily, I've participated in groupwork, it has helped. P1
Usually, I go through what has happened during the week. I attend because it helps me keep my problem in mind, so that it doesn't just diminish and diminish, and then, finally, I take that first beer. P8

One patient described how supportive and understanding his family had been about his situation.

They've understood me really well, there's been no schism. I'm quite happy about how they've taken it. P2

All interviewee groups stressed the importance of being drugfree.

I'm gonna do really well, as long as drugs and crime don't enter into the picture. P7

The interviewees maintained that successful forensic rehabilitation required that the index offense had been emotionally and cognitively integrated into the life story of the patient. This enabled the patient to focus on making concrete and realistic plans, and to move on in life. This, in turn, provided aspiration and structure.

I can ask him anything, and he can be, like, “I would never do that again,” and he regrets it so much and is aware of his illness and does not resist medication. N1

The interviewed nurses underlined the importance of practicing various life skills and self-control as rehabilitation became increasingly focused on life outside hospital. The patients emphasized the meaning of independent or semi-independent living conditions, supported employment, recreational hobbies, and close relationships.

To recover so that I can live independently, that is what I'm aiming for. And I'll go to work at the occupational center, have hobbies, see my family. P3

One carer emphasized the importance of meaningful activities, based on patients' individual skillsets and predilections.

At the hospital the therapist said that they have trouble finding him meaningful work, because of his high skill-level, particularly in metalwork. He said from the beginning that he always had aspirations, he was always making plans, for when he gets out. C3

## Discussion

Our research highlights three areas for development in forensic psychiatric services. First, it calls for increased risk awareness and risk assessment skills at the general psychiatric level. Second, it emphasizes the need for increased therapeutic engagement throughout the rehabilitative process. Third, it calls for structured and meaningful post-discharge aftercare. The results of this study are in line with Shepherd et al. (45) whose review emphasized safety and security as a necessary basis for the recovery process, the dynamics of hope and social networks in providing support and work on identity as an integral driver of change throughout the recovery process.

It has been estimated that as many as 75% of forensic patients have had contact with psychiatric services before their index offense (46), and that their hospitalizations prior to this offense have been longer than other patients' (47). Also, compared with other psychiatric patients, forensic patients have had more interruptions in education, as well as a higher degree of antisocial behavior (47, 48), social marginalization and childhood exposure to parental alcohol abuse (49). To develop services in line with the WHO action plan, pre-emptive interventions need to be made available at the most accessible, basic level in order to better serve the needs of this vulnerable group. Youth work, general medical and psychiatric services, and emergency units should also have access to specialist, individualized forensic consultations (50, 51). According to Kennedy (20), individual needs must be respected within forensic services. Processes for triage, allocation, and waiting-list management should be clearly defined to ensure that pathways function quickly in response to needs. Triage criteria should focus on the patient rather than the institution and should be described in meaningful units of difference or reliable change (20).

Bearing in mind the many life-long challenges forensic patients typically face, it is important to consider variables that not only relate the time before and during forensic treatment, but also to the follow-up period, in order to identify patients at risk of criminal recidivism (52). Our research partly echoes the work by Coid et al. (53), who identified causal risk factors for violence among discharged patients and argued that poor insight, symptoms of major mental disorder, poor treatment response, low level of personal support, and unsatisfactory living conditions all increased risk of violence, when accompanied by violent ideation, behavioral instability, and stress. Similarly, empathy, coping, work, leisure activities, good financial management, motivation for treatment, positive attitudes to authority, life goals, taking medication, and positive social networks all conveyed protective effects and reduced the risk of violence, but only when accompanied by good self-control. Indeed, as Simpson and Penney (54) and others (55) point out, bridging the concepts of security and therapy is perhaps most crucial to the recovery of the forensic patient.

Commitment to treatment measures may be promoted by recovery- and strength-based practices, including peer support (56, 57), opportunities for meaningful occupation (58), and work skills programs, such as Individual Placement Support (IPS) (59). Accordingly, Livingstone (60) concluded that success in the forensic mental health system is seen as a dynamic, collaborative process rather than an end state. Markham (61), too, calls for inclusive patient collaboration throughout all facets of personal recovery, including risk management. However, in order to ensure best recovery-oriented practices, forensic psychiatric services need to be developed at several levels.

As elsewhere (8, 62), through a process of transinstitutionalization, supported housing provision in Finland has increased (63). Despite this, the continued trend of deinstitutionalization combined with changes in mental healthcare funding and associated budget cuts (7) have resulted in an increase in social marginalization, untreated substance abuse, and psychiatric morbidity (64, 65). Similarly, De Page and Titeca (66) concluded in their analysis of 10 years' routine data collection that the severity in terms of risk, psychopathic personality traits, and lack of cognitive and functional capacities of forensic psychiatric patients had increased. Chow, Ajaz, and Priebe (67) qualitatively explored the perspectives of mental health professionals on what has driven the changes in institutionalized mental health care in Western Europe. They identified four major drivers of change: the overall philosophy of deinstitutionalization, with the aim to overcome old-fashioned asylum-style care; finances, with a pressure to limit expenditure and an interest of provider organizations to increase income; limitations of community mental health care, in which most severely ill patients may be neglected; and an emphasis on risk containment so that patients posing a risk may be cared for in institutions. In short, forensic services risk becoming increasingly burdened by patients previously cared for within a more robust general psychiatric service (10, 19, 68).

All in all, in line with the WHO action plan, our research calls for increased individuality when delivering all levels of service. Meaningful activities are necessary, such as supported work, maintaining freedom from substance misuse, enabling self-determination, and a participatory role whilst engaged in the services, bearing in mind the various aspects of security (20, 69–72). Thus, passive custodial institutionalization, or warehousing, must be replaced on an individual level by interactive and dynamic therapeutic security, supported by its procedural and physical counterparts, i.e., the institutional facets of security. Thus, high-quality forensic psychiatric services rely on appropriate hospital design, considering the typically long treatment periods and unique needs of many forensic patients (73).

We believe that the development and implementation of national quality standards, such as the gradated standards for low, medium (74), and high (75) security units in the UK, would be advisable within both Finnish institutional and community forensic mental health services (76). The upcoming reform of mental health legislation should consider the wider applicability of involuntary outpatient treatment, as suggested in a white paper commissioned by the Finnish Ministry of Social Affairs and Health (28). Development of forensic psychiatric services at all levels – including gradated security-aware standardization and patient triage – could help maintain an intact care pathway, to decrease offending, marginalization, and suffering. Only then can Finland begin to meet the requirements of the WHO Mental Health Action Plan for Europe.

## Strengths and Limitations of the Research

This was a qualitative study using a small sample recruited from two forensic psychiatric hospitals in Finland. The findings are considered to represent authentic experiences of eight forensic psychiatric patients, six parents of forensic psychiatric patients, and nine forensic psychiatric nurses. Our study has some limitations. We had a relatively small study sample, and the results may not be representative of all international forensic settings. However, our participants were experts in their area, with plenty of lived experiences.

## Conclusions and Future Directions

The results show that ensuring best recovery-oriented practices in forensic psychiatric services need to be developed at several levels. Our research calls for increased individuality when delivering forensic psychiatric services, within the context of gradated security-aware service standardization and patient triage. Risk awareness and risk assessment skills at the general psychiatric level should be increased and supported by forensic expertise in order to develop the continuum of national service provision. Non-responsiveness to treatment and substance use are linked to physical, procedural and relational security. Therefore, increased therapeutic engagement is required throughout the rehabilitative process. Structured and meaningful post-discharge aftercare needs to be developed. This calls for more diverse and meaningful activities and patient involvement. To close the identified gaps in services and to create gradated security-aware processes for patient treatment and triage, Finland needs to develop and implement national standards for forensic psychiatric care.

## Data Availability Statement

The original contributions presented in the study are included in the article/supplementary material, further inquiries can be directed to the corresponding author/s.

## Ethics Statement

The studies involving human participants were reviewed and approved by Ethics Committee of the Hospital District of Helsinki and Uusimaa. The patients/participants provided their written informed consent to participate in this study.

## Author Contributions

RA, OL, and AS contributed to the conception and design of the study and wrote the initial draft of the manuscript. RA acquired and analyzed the data and acted as the auditor of results and interpretation of data. All authors worked with the thematic analysis in joint meetings. Citations were translated by AS. All authors contributed to the article and approved the submitted version.

## Funding

This study was supported by Helsinki University Hospital, Psychiatry.

## Conflict of Interest

The authors declare that the research was conducted in the absence of any commercial or financial relationships that could be construed as a potential conflict of interest.

## Publisher's Note

All claims expressed in this article are solely those of the authors and do not necessarily represent those of their affiliated organizations, or those of the publisher, the editors and the reviewers. Any product that may be evaluated in this article, or claim that may be made by its manufacturer, is not guaranteed or endorsed by the publisher.
